# Resource Utilization for Patients With Distal Radius Fractures in a Pediatric Emergency Department

**DOI:** 10.1001/jamanetworkopen.2019.21202

**Published:** 2020-02-14

**Authors:** Keith J. Orland, Adam Boissonneault, Andrew M. Schwartz, Rahul Goel, Robert W. Bruce, Nicholas D. Fletcher

**Affiliations:** 1 Department of Orthopaedic Surgery, Emory University School of Medicine, Atlanta, Georgia; 2 Department of Orthopaedic Surgery, Children’s Healthcare of Atlanta, Atlanta, Georgia

## Abstract

**Importance:**

Improvement of clinician understanding of acceptable deformity in pediatric distal radius fractures is needed.

**Objective:**

To assess how often children younger than 10 years undergo a potentially unnecessary closed reduction using procedural sedation in the emergency department for distal radial metaphyseal fracture and the associated cost implications for these reduction procedures.

**Design, Setting, and Participants:**

This retrospective cross-sectional study included 258 consecutive children younger than 10 years who presented to a single, level I, pediatric emergency department and who had a distal radius fracture with or without ulna involvement between January 1, 2016, and December 31, 2017. Reductions were deemed to be potentially unnecessary if the coronal and sagittal plane angulation of the radius bone measured less than 20° and shortening measured less than 1 cm on initial injury radiographs. Use of procedural sedation or transfer status to another facility was noted if present. Statistical analysis was performed from April 2019 to June 2019.

**Main Outcomes and Measures:**

Potentially unnecessary reduction was the primary outcome. Radiographic findings were measured to determine reduction necessity. Additional variables measured were age, sex, time in the emergency department, transfer status, required reduction procedure, use of sedation, and cost associated with care.

**Results:**

Of the 258 participants studied, 156 (60%) were male, with a mean (SD) age of 6.7 (2.3) years. Among 142 patients (55%) who underwent closed reduction with procedural sedation in the emergency department, 38 (27%) procedures were determined to be potentially unnecessary. Review of *Common Procedural Terminology* charges revealed an approximately $7000 difference between the stated cost of a reduction procedure in the emergency department vs a cast application in an outpatient orthopedic clinic for distal radial metaphyseal fractures. The mean (SD) maximal angulation in either plane for fractures that underwent appropriate reduction was 30.6° (10.3°) compared with 13.9° (4.5°) for those unnecessarily reduced (*P* < .001). Patients who were transfers from other facilities were more than twice as likely to undergo a potentially unnecessary reduction (odds ratio, 2.3; 95% CI, 1.1-5.0; *P* = .03).

**Conclusions and Relevance:**

The findings suggest that improved awareness of these acceptable deformities in young children may be associated with limiting the number of children requiring reduction with sedation, improving emergency department efficiency, and substantially reducing health care costs.

## Key Points

**Question:**

How often do children younger than 10 years with distal radius fracture undergo a potentially unnecessary closed reduction with manipulation and procedural sedation?

**Findings:**

In this cross-sectional study of 258 children younger than 10 years with a distal radial metaphyseal fracture who were seen in a pediatric emergency department, 55% underwent closed reduction with procedural sedation; 27% of these were considered to be potentially unnecessary.

**Meaning:**

The findings suggest that improved awareness of remodeling potential and acceptable deformity for distal radial metaphyseal fractures in young children may be associated with improved emergency department efficiency, reduced health care cost, and reduced number of children undergoing closed reductions with sedation.

## Teaser

This cross-sectional study examines the frequency of potentially unnecessary closed reduction procedures using manipulation and procedural sedation in children with a distal radial metaphyseal fracture.

## Introduction

Fractures in children represent a substantial proportion of pediatric emergency department (ED) visits in the United States, with forearm fractures being the most common type of fracture (17.8%).^1^ Acute treatment pathways for pediatric forearm fractures generally include either splint or cast immobilization of the fracture in situ with orthopedic outpatient referral or closed reduction with splinting or casting through a variety of methods, such as hematoma blocks, bier blocks, or even procedural sedation. Although the latter treatment pathway is commonly performed, it is known to be associated with increased costs and complications.^2,3^

Closed reduction and casting of forearm fractures in the ED has been shown to cost significantly more than in situ immobilization in the ED and almost 5 times more than when performed as an outpatient in the pediatric orthopedic office.^2,4^ To assist in clinical decision-making regarding which fractures require a closed reduction procedure, multiple studies^5,6,7,8,9,10^ have defined acceptable parameters for fracture deformity that will remodel based on patient age, location of deformity, and degree of deformity. In general, larger angular deformities, particularly at the distal end of the forearm, are accepted for younger patients owing to their increased bone remodeling potential.^11,12,13,14,15^

**Figure 1.**
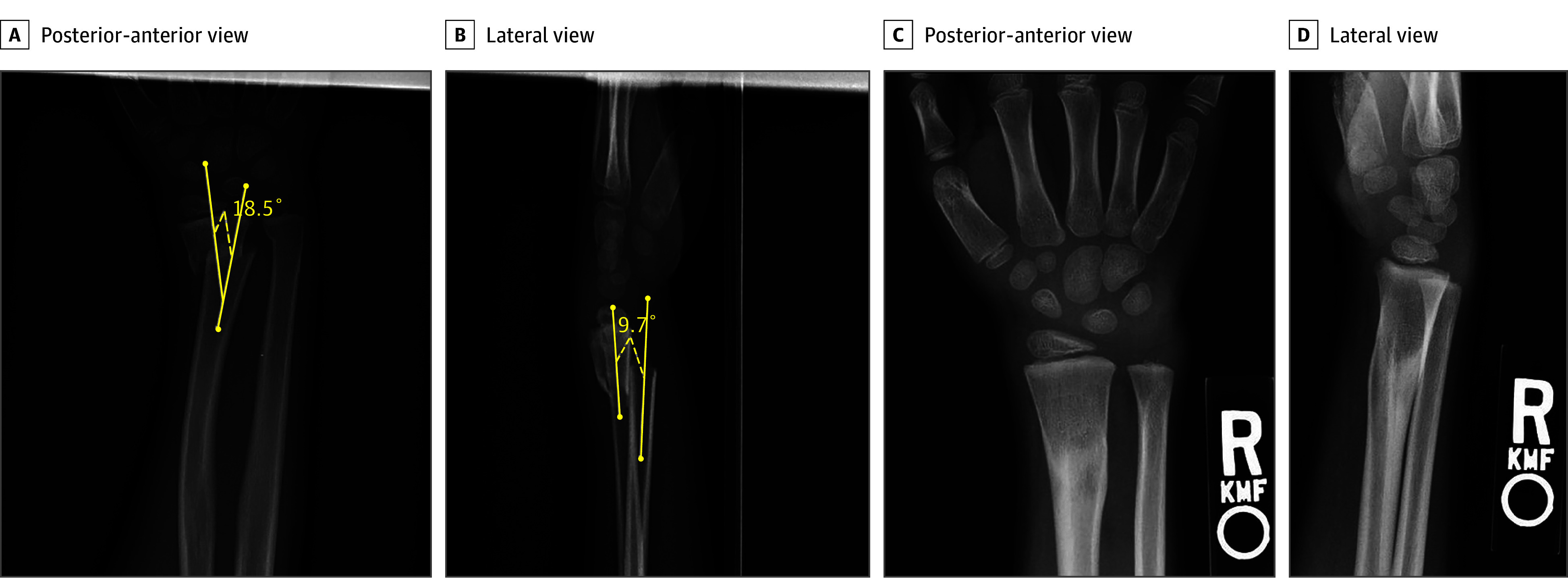
Remodeling Potential of Distal Radius Fracture in a Child Younger Than 10 Years

In children younger than 10 years, bayonet apposition (fractures with overriding fragments) of the distal radius with 100% displacement and less than 20° angulation may still be treated without any formal closed reduction and with excellent radiographic and functional outcomes (Figure 1).^2,5^ In 2012, Crawford et al^2^ reported excellent clinical and radiographic outcomes in 100% of patients treated within these guidelines. Despite these findings, the decision to tolerate a grossly visible deformity in a young child can be met with hesitation from patients, families, and ED clinicians.

We hypothesized that a significant number of children with distal radius fractures with acceptable alignment were undergoing potentially unnecessary reduction with procedural sedation despite the evidence supporting in situ immobilization. In addition, we hypothesized that there would be a correlation between patients who transfer from outside hospitals and the “overtreatment of” distal forearm fractures.

## Methods

This was a retrospective cross-sectional study of 558 consecutive patients seen at a single-institution pediatric ED between January 1, 2016, and December 31, 2017, for closed, distal radius metaphyseal fractures with or without an associated ulna fracture. Statistical analysis was performed from April 2019 to June 2019. Patients were captured for review based on the following *International Statistical Classification of Diseases and Related Health Problems, Tenth Revision (ICD-10*) diagnosis codes: S52.90XA, S52.91XA, S52.92XA, S52.591A, S52.592A, S52.531A, S52.532A, S52.541A, S52.542A, S52.561A, and S52.562A. Consistent with the policies of Emory University School of Medicine, Atlanta, Georgia, for studies using patient information, the present study was reviewed and accepted after an institutional review board evaluation. Owing to the retrospective nature, the study meets criteria for waiver of informed consent per the Emory University Institutional Review Board. This study followed the Strengthening the Reporting of Observational Studies in Epidemiology (STROBE) reporting guideline.

**Figure 2.**
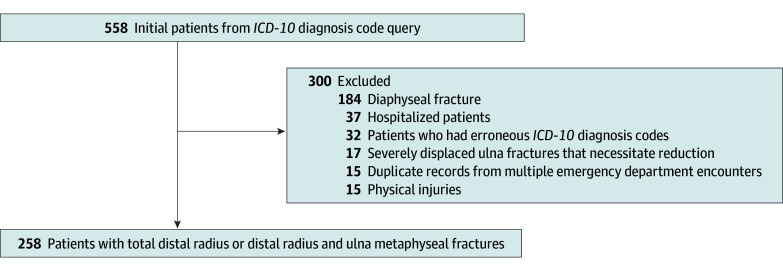
Study Flowchart *ICD-10* indicates *International Statistical Classification of Diseases and Related Health Problems, Tenth Revision*.

Inclusion criteria were age of younger than 10 years and presentation to the pediatric ED with an isolated distal radius metaphyseal fracture or both distal radius and ulna metaphyseal fractures. Exclusion criteria were hospital admission (n = 37) and presence of diaphyseal or proximal radius and/or ulna fractures (n = 184) or physeal injuries of the distal radius (n = 15). Severely displaced or angulated ulna fractures that required reduction independent of the radius fracture were also excluded from our analysis (n = 17). This was defined as an ulna fracture with shortening of more than 1 cm or angulation more than 20° in the sagittal or coronal plane. We also excluded any patients who were found to have improper diagnosis codes captured from our *ICD-10* diagnosis code query (n = 32). Any patients who had multiple ED encounters (n = 15) were only included once for statistical analysis (Figure 2).

Of the initial 558 patients, 258 were included for further statistical analysis. Clinical data were obtained from the patient electronic medical record and included age, sex, and primary payer status (private vs public). Total time in the ED was obtained by reviewing time stamps for admission and discharge from the pediatric ED. If a patient transferred from an outside facility or clinic, the transfer facility and county information were recorded. For children who underwent fluoroscopy-assisted closed reduction, analgesia type was recorded (bier block, hematoma block, or procedural sedation). These procedures were performed by the on-call orthopedic resident who was either a postgraduate year 3 or 4 resident at that institution. If the reduction was performed with procedural sedation, total sedation time was documented as well as the medications used for sedation and any associated adverse events that occurred during or after sedation. An adverse event was included for review if the event was documented within the sedation notation by the ED physician performing the sedation. A severe complication included hypoxia and/or an apneic event as defined by previous literature.^3^

**Figure 3.**
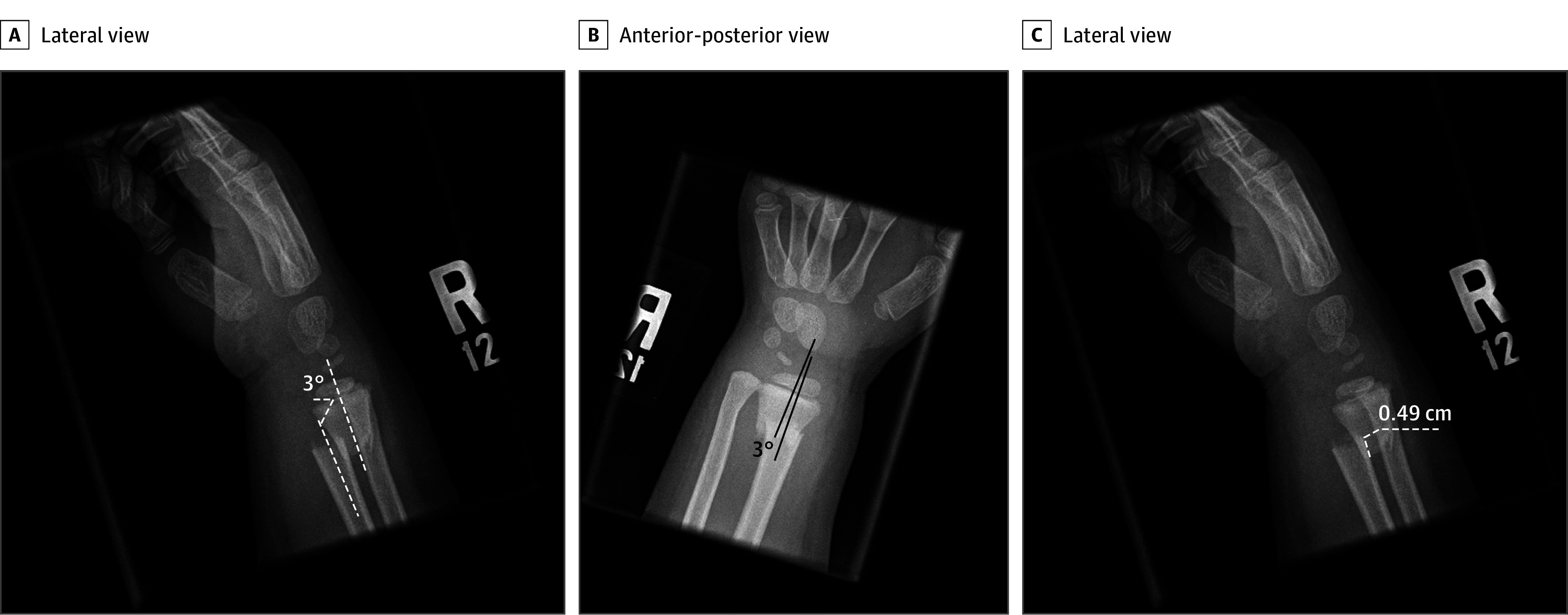
Radiographs of a Potentially Unnecessary Reduction

Radiographic assessment of the 258 distal radius fractures were analyzed by 3 of us (K.J.O., A.M.S., R.G.) using standard electronic tools available in the Picture Archiving Communication System (General Electric Company). Shortening, angulation, and the presence or absence of an ulna fracture were recorded for all fractures. Radiographs were randomly and evenly distributed among the 3 of us for review. Each author who performed measurements was blinded to outcomes data. We defined a closed reduction under procedural sedation as potentially unnecessary when the fracture had shortening less than 1 cm or angulation less than 20° in the sagittal and coronal planes, based on the literature^2,5^ (Figure 3).

Financial analysis for an episode of care of a distal radius fracture in the ED was completed with the use of *Common Procedural Terminology* (*CPT*) codes associated with closed reduction and manipulation procedures. We obtained institutional *CPT* code cost data for initial injury radiographs, intravenous medication for sedation, fracture care associated with manipulation, and the use of a portable fluoroscopy machine.

### Statistical Analysis

The distribution of continuous numerical data was examined in descriptive histograms and box plots, and a 2-sided Kolmogorov-Smirnov test was used to confirm a normal distribution. For descriptive analysis, absolute mean values for radiographic measurements were expressed in degrees and centimeters with SDs. Independent samples *t* tests were used to compare radiographic and clinical data between patients who underwent reductions and those who did not. Pearson χ^2^ tests (1-sided) were used for categorical data to determine the association of interinstitutional transferring with reduction. Univariate logistic regression analysis was performed to determine odds for a potentially unnecessary reduction dependent on transfer status. In all, 30 randomly selected radiographs were chosen to evaluate intraobserver and interobserver variability. To determine intraobserver variability, the same observer, blinded from the initial measurements, measured a random sampling of previously reviewed images after 4 weeks. For interobserver variability, a separate single observer repeated the measurements after a similar 4-week interval. A 2-way random-effects model for absolute agreement was performed, and intraclass correlation coefficients are reported. All statistical analyses were 2-sided with a statistical significance of *P* < .05, and were performed with Stata, version 14 (StataCorp LLC).

## Results

Of the 258 included patients, 156 were male (60%), with a mean (SD) age of 6.7 (2.3) years. For payer types, 113 (44%) were privately insured and 145 (56%) had Medicaid or were uninsured. Of the 258 distal radius fractures, 187 (72%) had an associated ulna fracture. Ulna fractures ranged in severity from buckle to completely displaced fractures. The overall rate of children who underwent procedural sedation in the ED was 55% (142 of 258). The mean (SD) maximal angulation (coronal or sagittal plane) was 26.2° (11.7°) for fractures that were reduced compared with 9.1° (8.2°) for those that were not reduced (*P* < .001). Intraobserver reliability for radiographic measurement of shortening was 0.985 (95% CI, 0.969-0.993) and of angulation was 0.993 (95% CI, 0.968-0.997) using an intraclass correlation coefficient. Interobserver reliability was equally as strong for radiographic measurements for shortening at 0.987 (95% CI, 0.973-0.994) and for angulation at 0.988 (95% CI, 0.975-0.994). There was a significant difference in time spent in the ED for children who underwent closed reduction (mean [SD], 4.3 [1.2] hours) compared with those who did not (mean [SD], 2.2 [1.3] hours) (*P* < .001).

The overall rate of transfer from an outside facility was 24% (61 of 258 children). The rate of reduction for patients who were transferred from an outside facility and who underwent reduction using procedural sedation was 93% (57 of 61). In contrast, 43% (85 of 197) of patients who were not transferred underwent a reduction with procedural sedation (*P* < .001) (Table 1). There was no significant correlation between patient insurance status and transfer from an outside facility.

**Table 1.** Reduced vs Nonreduced Distal Radius Fracture Characteristics and Association With Transfer Status

Characteristic	Distal Radius Fracture		*P* Value
	Reduced (n = 142)	Nonreduced (n = 116)	
Time in emergency department, mean (SD), h	4.3 (1.2)	2.2 (1.3)	<.001
Maximum angulation, mean (SD), °	26.2 (11.7)	9.1 (8.2)	<.001
Transferred, No./total No. (%)			
Yes	57/61 (93)	4/61 (7)	<.001^a^
No	85/197 (43)	112/197 (57)	

^a^ Rate of reduction for comparison between transferred and nontransferred patients.

When considering only patients who underwent closed reduction with procedural sedation in the ED, 38 of 142 (27%) were determined to have undergone potentially unnecessary reductions. The mean (SD) maximum angulation of the radius in either plane for fractures that underwent appropriate reduction was 30.6° (10.3°) compared with 13.9°(4.5°) for fractures that were unnecessarily reduced (*P* < .001). There was no significant difference in mean (SD) shortening of distal radius fractures between those appropriately reduced (0.3 [0.5] cm) and those potentially unnecessarily reduced (0.4 [0.5] cm) (*P* = .31). Of the 38 patients who underwent a potentially avoidable reduction, 21 (55%) had been transferred from an outside facility. In a logistic regression model, patients who had been transferred from another facility were more than twice as likely to undergo a potentially unnecessary reduction (OR, 2.3; 95% CI, 1.1-5.0; *P* = .03). Among transferred patients who underwent a closed reduction, the potentially unnecessary reduction rate was 37% (21 of 57) compared with 20% (17 of 85) among nontransferred patients who underwent a closed reduction (*P* = .03) (Table 2). Of the 116 distal radius fractures that did not undergo a closed reduction in the ED, 7.8% (9 of 116) met criteria for a closed reduction in the ED.

**Table 2.** Appropriate vs Potentially Unnecessary Reduction Characteristics and Association With Transfer Status

Characteristic	Reduction Procedure		Odds Ratio (95% CI)	*P* Value
	Appropriate (n = 104)	Potentially Unnecessary (n = 38)		
Maximum angulation, mean (SD), °	30.6 (10.3)	13.9 (4.5)	NA	<.001
Shortening, mean (SD), cm	26.2 (11.7)	0.3 (0.5)	NA	.31
Transferred, No./total No. (%)				
Yes	36/57 (63)	21/57 (37)	2.3 (1.1-5.0)^a^	.03
No	68/85 (80)	17/85 (20)		

Abbreviation: NA, not applicable.

^a^ Odds ratio for comparison of likelihood of potentially unnecessary reduction for transferred vs nontransferred patients.

We also investigated the events associated with procedural sedation among children who underwent closed reductions. Total mean (SD) sedation time was 13.9 (9.2) minutes. The overall sedation complication rate in the cohort was 14.8% (21 of 142). Adverse events included hypoxia or an apnea event requiring airway support (n = 10), secretions requiring suction (n = 1), intrasedation rash treated with intravenous diphenhydramine (n = 1), myoclonus (n = 1), and postsedation nausea or emesis (n = 8). There was no statistically significant difference in overall complication rates between patients who underwent appropriate reductions (15.4% [16 of 104]) and those who underwent potentially unnecessary reductions (13.2% [5 of 38]) (*P* = .74). Severe complications in the cohort included apnea events and hypoxia as recorded by the ED clinician’s document when a patient required supplemental oxygen during the sedation or required bag mask ventilation. The rate of severe complications among the patients was 7.0% (10 of 142). No patients with reported adverse events required admission to the hospital secondary to the recorded complication.

Institutional financial review of the *CPT* code charges for closed reduction and manipulation using procedural sedation in the ED showed a cost of $8077.50. This cost included initial radiographs of the forearm and wrist, sedation medication and monitoring, use of a portable fluoroscopy machine for reduction, and closed reduction and fracture care. This result was approximately 8 times as costly compared with previously published costs of outpatient cast immobilization *CPT* code charges ($1027).^2^

## Discussion

Distal radius fractures of the forearm are the most common fracture type, representing nearly 25% to 30% of all fractures in children.^16,17^ On the basis of epidemiologic estimates for children younger than 10 years, the incidence of these fractures has been increasing over time, and they are estimated to represent approximately 280 000 ED visits yearly.^1,17,18,19,20,21,22,23^ In a review article on distal radius and forearm fractures in children, Noonan and Price^5^ described that bayonet apposition and up to 20° angulation is acceptable for distal radius fractures among children with at least 2 years of growth remaining. Crawford et al^2^ confirmed this remodeling potential of displaced and angulated distal radius fractures with a series of 51 children younger than 10 years, all of whom had excellent functional and radiographic outcomes with closed treatment without reduction.^2^ They showed in their series that obtaining gentle correction of angulation to 10° or less without analgesia was not difficult during outpatient cast application. In a similar study by Do et al,^4^ 100% of patients in their series had no significant clinical deformity or residual functional deficit when redisplaced distal radius fractures were treated without remanipulation.

Our study showed that 27% of all distal radius fractures that underwent closed reduction with sedation in our designated level I pediatric ED could have potentially been managed with in situ immobilization and gentle correction in office in accordance with the study by Crawford et al.^2^ This alternative would have reduced the family’s and patient’s ED visit by nearly 2 hours. In addition, choosing the alternative may improve the ED workflow by reducing the personnel and resources required to perform sedation. For our institution, this workflow change equates to 2 nurses, an ED resident, an ED faculty physician, an orthopedic technologist, and an orthopedic resident. The mean (SD) angulation for potentially unnecessarily reduced distal radius fractures in our series was 13.9° (4.5°). This result falls within the limits of the defined safe criteria in the series by Do et al^4^ that specified that the fracture should heal without any clinical or functional deformity with remodeling. With reported rates of redisplacement from 20% to 30% despite anatomic manipulation at presentation, reinforcing remodeling potential alone for these fractures may avoid what is often a temporary reduction and may eliminate the need to closely monitor redisplacement to initial deformity.^2,24,25,26,27,28,29,30,31,32^ With remodeling potential of approximately 2.5° per month, these injuries have near anatomic position after approximately 6 months of growth.^33^

Manipulation under procedural sedation is associated with both significant benefit and risk. Although intravenous sedation is effective for pain control and muscle relaxation, several reports^3,34,35,36,37^ have shown the rate of adverse events associated with sedation to be approximately 15%, with the rate of serious complications, including hypoxia and stridor, to be as high as 12%. Our study results agree with the previous literature,^3,34,35,36,37^ with a total cohort sedation complication rate of 14.8% and with 7% of complications qualifying as serious events requiring additional treatment and observation in the ED.

In addition to decreasing total time spent in the ED and to reducing the risks associated with manipulation and sedation, a shift in this acute treatment algorithm has significant potential cost savings for health care payers and the health care system in general. Institutional financial analysis revealed the cost for a closed reduction and manipulation using procedural sedation in our ED to be $8077.50. This cost is similar to previously published data^38^ on average cost of ED fracture management of $7000. Significant cost savings have been shown when comparing closed reduction and manipulation in the ED with splinting and outpatient referral for moderately displaced distal radius fractures.^2,4,39^ Our study showed nearly an 8-fold increase in cost when comparing previously reported costs of outpatient cast immobilization compared with closed reduction and procedural sedation in the ED. Of all presenting distal radius fractures in our series, 15% (38 of 258) were deemed to be potentially unnecessary. With an estimated 280 000 distal radius fractures per year among children younger than 10 years seen in EDs across the United States, this would equate to potential health care savings of nearly $270 million, based on a 15% unindicated reduction rate. Although this figure is just an estimate, it is indicative of the profound cost associated with these highly incident fractures. With improved treatment algorithms, there is opportunity for cost reduction without sacrificing clinical outcomes.

Our institution is a large, tertiary referral, pediatric hospital in a large metropolitan city. Transfers from outside institutions to our facility are not uncommon since it is a nonprofit academic institution with a vast array of subspecialists. In general, patients with government or no insurance or those requiring subspecialist or higher acuity care have high rates of transfer from 1 facility to another.^40^ Our data showed no significant difference in transfer rates based on type of payer. There was a significant difference in the rate of reductions for children who were transferred (93%) compared with those not transferred (43%). Although it might be expected that transferred patients would have a higher incidence of closed reduction after the staff at the transferring institution had already considered in situ immobilization, children transferred were twice as likely to undergo a potentially unnecessary reduction. To our knowledge, this is the first study identifying rates of reduction for patients that transfer for fracture care. The discrepancy in overall reduction rates and rates of potentially unnecessary reductions is likely in part attributable to the expectation created by staff as well as the family of a patient with a displaced fracture. At this time, we recommend that a telephone consultation with an on-call orthopedic physician before transfer be made to potentially save significant time, cost, and hospital resources.

With distal radius fractures representing one of the most common causes for pediatric ED visits in the United States, adhering to the well-published remodeling potential of pediatric distal radius fractures boasts significant opportunity to provide cost-effective fracture care. Whereas our data suggested a profound benefit to the health care system, it was likely a conservative measure. For institutions without full-time availability of sedation for reduction in the ED, the costs of hospital admission and reduction in the operating room are likely even greater. The unrealized cost and patient inconvenience of emergency vehicle transfer and time spent at a second ED were not included in our study, but likely represent a substantial monetary figure. Improved education with radiographs at various time points showing the remodeling potential of angular deformity and shortening would be helpful for clinicians and families. Our findings support the need for interdisciplinary and nationwide awareness of the data that supports strict criteria for closed reduction of the common pediatric distal radius fracture.

### Limitations

This study has limitations. The retrospective design of this study at a single center is a limitation. We obtained the patient data set using a query of common *ICD-10* diagnosis codes. However, this was likely not exhaustive and may not have captured all patients with distal radius fractures who presented to our institution during this period owing to miscoding. We found that 32 patients were errantly included in the initial cohort during the filtering process, which suggests the potential for unintentional exclusionary miscoding not captured in this review. Second, there was potential abstraction bias and nonobjective evaluation of radiographs among us. We attempted to mitigate this bias by the random, equal assignment of radiographs for the 3 reviewers to interpret, with the reviewers being blinded to the outcome data. In addition, a blinded post hoc analysis to determine interobserver and intraobserver reliability confirmed absolute agreement. Moreover, the decision of potentially unnecessary reduction was made solely based on radiographic measurements obtained from satisfactory radiographs used for interpretation at the time of injury. for interpretation. In addition, our institution’s ED coding and billing may vary compared with other institutions and thus would alter the estimated values that we presented.

## Conclusions

Our data suggest that a significant number of children presenting to our ED with distal radius fractures could have had the fractures immobilized and treated as an outpatient with clinical cast application instead of having undergone procedural sedation and manipulation, based on evidence-driven radiographic and demographic criteria. The findings suggest that improved awareness of these acceptable deformities in young children may be associated with limiting the number of children requiring reduction with sedation, improving emergency department efficiency, and significantly reducing health care costs.
